# Effect of *Lactobacillus plantarum* P9 on defecation, quality of life and gut microbiome in individuals with chronic diarrhoea: Protocol for a randomized, double-blind, placebo-controlled clinical trial

**DOI:** 10.1016/j.conctc.2023.101085

**Published:** 2023-02-01

**Authors:** Wenjun Liu, Nong-Hua Lu, Xu Zhou, Yingmeng Li, Yong Xie, Longjin Zheng, Weifeng Zhu, Qiuping Xiao, Ni Yang, Kexuan Zuo, Tielong Xu, Heping Zhang

**Affiliations:** a Evidence Based Medicine Research Center, Jiangxi University of Chinese Medicine, Nanchang, 330004, China; b Department of Gastroenterology, The First Affiliated Hospital of Nanchang University, Nanchang, 330006, China; c Key Laboratory of Dairy Biotechnology and Engineering, Ministry of Education, Key Laboratory of Dairy Products Processing, Ministry of Agriculture and Rural Affairs, Inner Mongolia Key Laboratory of Dairy Biotechnology and Engineering, Inner Mongolia Agricultural University, Hohhot, 010018, China; d State Key Laboratory of Innovative Medicines and High-efficiency Energy-saving Pharmaceutical Equipment, Nanchang, 330006, China

**Keywords:** Chronic diarrhoea, Probiotics, Effectiveness, Clinical trial, *Lactobacillus plantarum*

## Abstract

**Background:**

Probiotics may be an ideal choice for these patients, given it can improve the defecation and quality of life of individuals with chronic diarrhoea. However, evidence-based medical research is still limited to support its use as a diarrhoea agent.

**Method:**

A randomized, double-blind, placebo-controlled clinical trial is designed to pinpoint the efficiency and possible action modes of probiotics for chronic diarrhoea. 200 eligible volunteers with chronic diarrhoea are randomly assigned to a probiotic group (orally taking *Lactobacillus plantarum* p9 probiotics powder) or a placebo group. Except an independent project administrator who will be responsible for unblinding, the other researchers are blinded. Primary outcome is diarrhoea severity score, and secondary outcomes include weekly mean frequency of defecation, weekly mean stool appearance score, weekly mean stool urgency score, emotional state score, gut microbiome, and faecal metabolome. Each outcome measure will be assessed at the timepoints of pre-administration (day 0), administration (day 14 and/or 28), and post-administration (day 42) to identity inter- and intra-groups differences. Adverse events will be recorded to evaluate the safety of *L. plantarum* p9.

**Discussion:**

The study protocol will provide high-quality evidence for the use of probiotics as a diarrhoea agent when it is strictly conducted out, providing evidence regarding whether and to what extent *L. plantarum* p9 can improve the defecation and well-being of individuals with chronic diarrhoea.

**Trial registration:**

Chinese Clinical Trial Registry (ChiCTR) (NO. ChiCTR2000038410). Registered on November 22, 2020, https://www.chictr.org.cn/showproj.aspx?proj=56542.

## 1 Background

Diarrhoea is defined as the situation that the stool weight is more than 200 g per 24 h containing more than 200 ml fluid per 24 h, or the times of defecating loose stools are greater than 3 within 24 h [[1], [2], [3]]. And an individual can be diagnosed as chronic diarrhoea when diarrhoea lasts more than 4 weeks [4,5]. The prevalence of chronic diarrhoea is estimated to be 3–5% among general population [6,7] and 9.6–14.2% in the elderly (aged more than 60) [8,9]. It may disturb the patient's quality of life, work performance and well-being as well as increase their medical expenses [10], and the chronic diarrhoea-related economical loss just from work-loss is estimated to be approximate $350, 000, 000 annually [11].

Probiotics always hold the advantages of safety and widely public acceptance [[27], [28], [29]]. Evidence has showed that probiotics are safe for the study volunteers across the whole age spectrum, only with caution advised in immunologically vulnerable populations [27], and without significantly side effects when used in the long-term for chronic diarrhoea [26]. However, data on the effectiveness of probiotics in the treatment of chronic diarrhoea is still lacking and limited to anecdotal experience shared by people [26,30] or case reports [31] which suggest that probiotics may offer a way to bring about resolution in chronic diarrhoea [26,30,31]. Thus, a study protocol for high-level evidence is warrant for the efficacy and safety of probiotics in individuals’ chronic diarrhoea.

## 2 Methods and design

### 2.1 Design

**Fig. 1:**
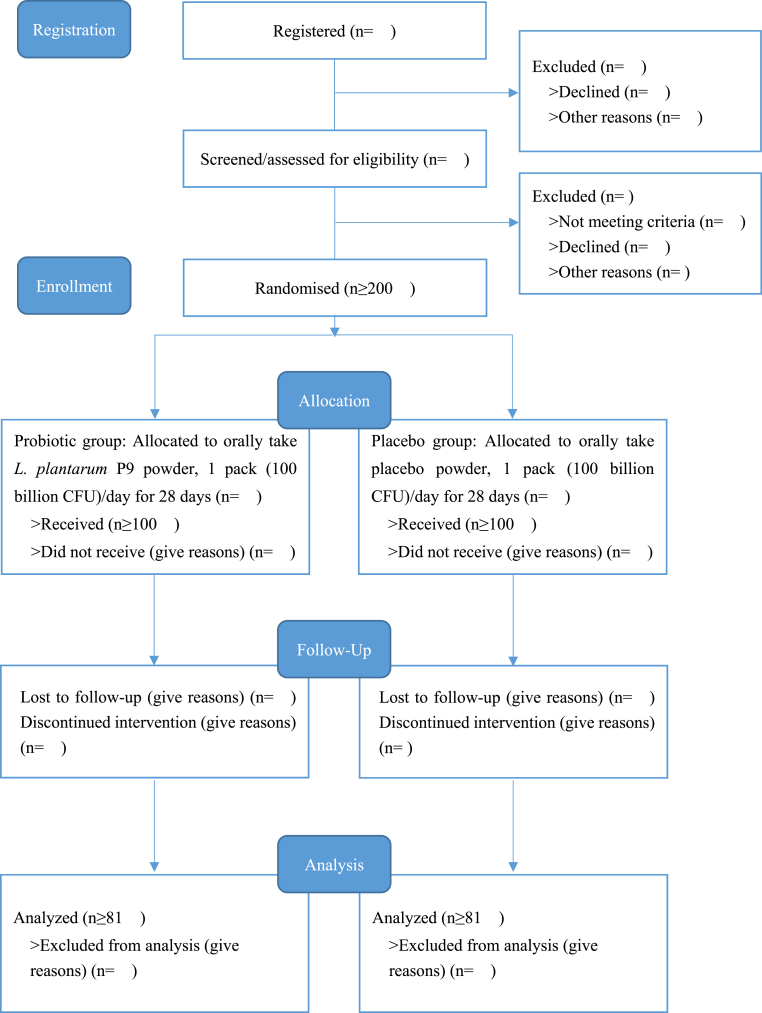
Flow chart of the protocol.

This study is a randomized, double-blind, placebo-controlled clinical trial, implemented in Nanchang city, Jiangxi province, China. Volunteers can orally intake *L. plantarum* p9 or placebo at home and are followed up online via completing designed online questionnaires. For the stool sample collection, volunteers are required to immediately inform researchers as soon as prepared according to instructions, and the researchers timely take it back for further detection. This paper is written according to the Standard Protocol Items: Recommendations for Interventional Trials (SPIRIT) [32]. The flow chart of the study is presented as Fig. 1.

### 2.2 Inclusion criteria

Eligible volunteers should have diarrhoea onset for at least 6 months before enrolment, with loose or watery stool (looked like Bristol type 5, 6, or 7 (Appendix 1) [33]) at least 25% of the times of defecation within the past 3 months. The volunteers involved in this study will be patients with chronic diarrhoea aged 18–65 years. For patients aged from 18 (exclusive) to 50 (inclusive) years, the result of stool test (including occult blood) conducted during the screening period is normal or is abnormal but is judged by the investigators as clinically irrelevant. For patients aged from 50 (exclusive) to 65 (inclusive) years, the result of colonoscopy performed at a tertiary or higher-level hospital within the past 6 months is normal or is abnormal but must be judged by the investigators as clinically irrelevant. Of course, the volunteers should be willing to sign the informed consent form, and only the written informed consent form is provided can then the volunteer participates in the trials.

### 2.3 Exclusion criteria

Volunteers have one of the following situations are excluded.

(1) Personal or family history of colon cancer, celiac disease, or inflammatory bowel disease.
(2) Confirmed Intestinal organic diseases by colonoscopy.
(3) Plans to be pregnant with or father a child in near future, or at the status of pregnancy or breastfeeding in women.
(4) Allergies to L. *plantarum* p9 or placebo.
(5) Intaking antibiotics or probiotics within the past two weeks.
(6) Intaking antianxiety, antidepressant, or other psychotropic drugs within the past month.
(7) Need for long-term use of medications for diarrhoea.
(8) History of severe diseases, such as myocardial infarction, cerebral infarction, and malignant tumour, judged by the investigators as disqualifying conditions.
(9) Major mental illnesses, inability to control one's actions, or inability to cooperate.
(10) Illiteracy, inability to understand the informed consent form, or inability to independently sign the informed consent form.

### 2.4 Volunteer recruitment

The investigators recruit volunteers from the public via oral communication, posters, and dissemination of WeChat official account. With the help of WeChat, a two-dimensional (QR) code is available for volunteers to scan and register for enrolment, submitting demographic data, and answering questions related to inclusion and exclusion criteria. Then, the potentially eligible volunteers will undergo three rounds of screening, as follows.

First, the registration information is collected and collated, according to which potentially eligible candidates are invited to participate in a face-to-face consultation with clinicians and researchers for the further screening.

Second, a series of face-to-face consultations are organized: 1) Trained researchers introduce the trial to the potentially eligible volunteers through video and information sheets regarding the main aspects of the trial, especially the content of informed consent materials, and discuss the trial with the potentially eligible volunteers. 2) Then, the clinicians also have an informed face-to-face consultation with the potentially eligible volunteers to confirm the registered information, further selecting out potentially eligible volunteers according to inclusion and exclusion criteria to proceed in the next procedure. The selected potentially eligible volunteers are essential to sign an informed consent form directly with a member of data management team (DMT) before proceeding into the next procedure.

Third, the potentially eligible volunteers undergo a 14-day screening period. During the third round of selecting, the potentially eligible volunteers are not allowed to take any medicines or health products to improve their diarrhoea symptoms. And each volunteer will be asked to collect one stool sample and complete an online defecation diary (Appendix 2). At the end of the screening period, the diary and stool exam results will be reviewed and used to select eligible volunteers. Only then, the selected eligible volunteers can take part in the following formal intervention and follow-ups (Fig. 1).

### 2.5 Randomization and blinding

Based on the order of registering during above recruitment, eligible volunteers are given a serially unique number, i.e., 001, 002, 003, 004, 005, and so on. The unique number is used as volunteer's identification card throughout the whole research periods instead of volunteer name or any other information, so as to guarantee confidentiality for volunteers. For each unique number, a random sequence is automatically generated using the computer software by two independent project administrators. The unique number with an odd random sequence is assigned into probiotic group, while the unique number with an even random sequence is assigned into placebo group. During the study, except two independent project administrators, the other participants of the study, including volunteers, treatment packs distributors, data collectors and analysts are blinded to the randomization sequence, namely the grouping. The randomization sequence is maintained by two independent project administrators and will only be unblinded in the case of major safety issues or when performing the interim or final data analysis. Moreover, the probiotics or placebo treatment packs are labelled with the foregoing unique numbers according to the grouping of the unique numbers, and the treatment packs distributors distribute the treatment packs according to the unique number on the packs to the corresponding volunteers, in such a way, allocation concealment is achieved.

### 2.6 Study interventions

One probiotics or placebo powder pack per day. One probiotics powder pack contains 100 billion colony-forming units (CFU) of *L. plantarum* P9. *L. plantarum* P9 powder comprises *L. plantarum* P9 (40%), maltodextrin (20%), orange powder (20%), and maltitol (20%). The placebo comprises maltodextrin (60%), orange powder (20%), and maltitol (20%) and has the same appearance, packaging, and taste as the *L. plantarum* P9 powder. Both probiotics and placebo packs are stored in a 4 °C, dry place away from direct sunlight. At the end of the phase, all unused probiotics or placebo and used (empty) packs are collected, so as to calculate volunteers’ compliance.

The study is designed to include three phases, a period of screening (an observation period of pre-administrating), an observation period of administrating, and an observation period of post-administrating (Table 1). The interventions in each phase are as follows.

(1) Period of screening (observation period of pre-administrating) (days −14 to 0): volunteers do not receive probiotics or placebo. Eligibility screening are performed as described in “volunteer recruitment part” (Table 1).
(2) Observation period of administrating (days 0–28): 1) For probiotics group: volunteers take *L. plantarum* P9 powder with warm boiled water (below 40 °C) on a full stomach, 1 pack per day (i.e., 100 CFU/d); if volunteers have to take antibiotics during the administration, the probiotics is taken 2 h later. 2) For placebo group: volunteers take the placebo in the same manner as probiotics group.
(3) Observation period of post-administrating (days 29–42): No interventions are taken during the study.

**Table 1:** Study and follow up schedule.

Item		Period of screening (observation period of pre-administrating)		Observation period of administrating		Observation period of post-administrating
		Visit 0	Visit 1	Visit 2	Visit 3	Visit 4
		(Days −14 to −1)	Day 0	Day 14	Day 28	Day 42
Collection of volunteers' basic information		**✓**				
Screening		**✓**				
Informed consent form		**✓**				
Stool test (including occult blood)		**✓**				
Defecation diary		Completed Online Daily				
Primary outcomes	Diarrhoea severity score		**✓**	**✓**	**✓**	**✓**
Secondary outcomes	Weekly mean frequency of defecation		**✓**	**✓**	**✓**	**✓**
	Weekly mean stool appearance score		**✓**	**✓**	**✓**	**✓**
	Weekly mean stool urgency score		**✓**	**✓**	**✓**	**✓**
	Emotional state score		**✓**	**✓**	**✓**	**✓**
	Gut microbiome		**✓**		**✓**	**✓**
	Faecal metabolome		**✓**		**✓**	**✓**
Safety measures	Adverse events (AEs)			**✓**	**✓**	**✓**
	Serious adverse events (SAEs)			**✓**	**✓**	**✓**
Verification of compliance with the intervention					**✓**	
Special situations related to defecation (e.g., concomitant medications)			**✓**	**✓**	**✓**	**✓**

Note: The outcome measures, weekly mean frequency of defecation, weekly mean stool appearance score and weekly mean stool urgency score are calculated from the defecation diary. The defecation diary completed from day −14 to −1 will be used for volunteer selection and baseline data. The detection of gut microbiome will be conducted within 2 months after the end of follow-ups, and the detection of faecal metabolome will be implemented within 2 months when data analysis finds that the probiotics present significant effect on gut microbiome.

### 2.7 Prohibited confounding interventions

The following interventions or factors may confound the results of the study and are prohibited: 1) other kinds of probiotics, prebiotics, and foods containing probiotics (such as yoghurt); 2) antianxiety, antidepressant, and other psychotropic drugs; 3) other substances may benefit intestinal symptoms. Furthermore, antibiotics are not advised, and normal dietary habits are recommended during the study. All concomitantly used substances/drugs related to defecation are required to record with an explanation via online daily diary (Appendix 2).

### 2.8 Primary outcome

The primary outcome is the diarrhoea severity score, which is set as to the gastrointestinal symptom rating scale (GSRS) [[33], [34], [35], [36]]. The outcome measure is equal to the sum of mean defecation frequency score per day, mean stool consistency score per time, and mean stool urgency score per time within the past 7 days (see Appendix 2). The diarrhoea severity score will be assessed on days 0 (baseline), 14, 28, and 42. A higher score indicates more severe diarrhoea, Table 1. The details of calculation are as follows.

A defecation diary developed with reference to the literatures [33,34,37] will be completed daily online by the volunteers (Appendix 2). Based on the diary, the mean defecation frequency score per day, mean stool consistency score per time, and mean stool urgency score per time will be obtained. The defecation frequency score is ranked as: 0-point, one to two a day; 1-point, three to four times a day; 2-point, five to six times a day; 3-point, seven times a day or more frequently. The mean defecation frequency score per day is equal to “total defecation frequency score within the past 7 days/7”. The stool appearance score refers to Bristol stool type (Appendix 1) and must be recorded for each defecation: types 4–7 correspond to 0–3 points, respectively. The mean stool consistency score per time is equal to “total stool consistency scores within the past 7 days/total times”. The stool urgency score must be also recorded for each defecation: 0-point, no urgency; 1-point, a little urgency; 2-point, sudden need to have a defecation that must be addressed immediately; or 3-point, incontinence resulting in uncontrolled defecation and may stain pants. The mean stool urgency score per time is equal to “total stool urgency score within the past 7 days/total times”.

### 2.9 Secondary outcomes

The secondary outcome measures include weekly mean frequency of defecation, weekly mean stool appearance score, weekly mean stool urgency score, emotional state score, gut microbiome, and faecal metabolome.

(1) Similarly, based on the diary (Appendix 2), the weekly mean frequency of defecation, the weekly mean stool appearance score, and the weekly mean stool urgency score within the past two weeks on day 0, day 14, day 28 to day 42 will be evaluated. It is noted that the weekly mean frequency of defecation, weekly mean stool appearance score, and the weekly mean stool urgency score on day 0 are obtained from the diary completed during screening period, Table 1.
(2) The Depression, Anxiety and Stress Questionnaire (DASS-21) [38] (Appendix 3) will be completed online on day 0 (baseline), day 14, day 28, and day 42, and emotional state score calculated from DASS-21 will be used to assess the volunteers' emotional status, Table 1.
(3) For the indicator of gut microbiome, DNA are extracted from stool sample using the QIAamp Fast DNA Stool Mini Kit (Qiagen, Hilden, Germany). An agarose gel electrophoresis and Nanodrop spectrophotometer are used to examine the quality of DNA. Then, shotgun metagenomic sequencing are performed for all samples using an Illumina HiSeq 2500 instrument. And sequence libraries are constructed by DNA fragments of ∼300 bp length which obtained from paired-end reads of ∼150 bp lengths generated by sequencing in bi-directions. In addition, the metagenomic analysis is performed from the following aspects: analysing alpha diversity and beta diversity in each group to determine the intra-groups differences in the microbiota compositions; analysing the inter- and intra-groups taxonomic characteristics at the levels of phylum, genus, and species to identify specific genes related to diarrhoea. Metagenomic biological pathway analysis are used to evaluate the effect of probiotics on the function of the gut metagenomics in patients with diarrhoea and hopefully to explore the metagenomic biological pathways contributing to the mechanism of probiotics on the treatment of diarrhoea.
(4) For the indicator of faecal metabolome, briefly, stool samples treated by protein precipitation method and the supernatant are used for liquid chromatography tandem mass spectrometry (LC-MS/MS) analysis. The original data is subjected to peak alignment, retention time correction and peak area extraction through the XCMS-Plus program. The structure of metabolites is identified by accurate mass matching (<5 ppm) and two-level spectrum matching, and METLIN database is retrieved. After deleting the missing value > 50% and normalizing the data, multi-dimensional statistical analysis is performed, like unsupervised principal component analysis (PCA), supervised partial least squares discriminant analysis (PLS-DA) and potential difference metabolite analysis. Metabonomic analysis can further identify the potential differential metabolites of probiotics in treatment of diarrhoea and examine the correlation analysis between the gut microbiota and metabolites.

### 2.10 Safety evaluation

Existed evidence shows that probiotics is safe, without significant adverse event (AE) compared with placebo group. Nevertheless, in the present study, four kinds of symptoms possibly related to probiotics are listed as possible AEs in the study, including symptoms may result from systemic infections, deleterious metabolic activities, excessive immune stimulation, and gastrointestinal side effects [39]. And severe AEs are those AEs result in volunteer's withdrawal, like hospitalization, disability, mortal danger, or death. When an AE occurs, the symptoms, time of onset, duration, causal relationship with interventions, and measures taken, will be accurately recorded during the study. Any serious AE will be recorded according to the corresponding criteria and emergency measures and promptly reported to the medical ethics committee of the first affiliated hospital of Nanchang university within 24 h, in addition, a separate AE report will be prepared. Judged from the AEs observed in the study, the safety of *L. plantarum* P9 powder will be evaluated as the following four levels: excellent (safe with no AE); good (relatively safe with moderate AEs that disappear on their own without any specific treatment); conditional (AEs that resolve after adopting certain measures and do not lead to study withdrawal); and unsafe (AEs that result in volunteer's withdrawal) [40].

### 2.11 Additional assessment

Demographic profiles, special situations related to defecation during the study, including change of dietary habits (e.g., eating spicy or oily foods or drinking), taking antibiotics and any other reported situation are recorded and assessed as needed.

### 2.12 Compliance monitoring and withdrawn

The L. *plantarum* P9 and placebo powder are supplied as treatment packs and packaged in boxes. At the beginning of administration phase, the probiotics or placebo packaging boxes enough for 28-day intervention are distributed to corresponding volunteers. Volunteers are required to keep all the used (empty) and unused treatment packs which are collected to check whether a volunteer takes the probiotics or placebo as the study's schedule. Based on the number of empty packs, the compliance rate is calculated for individual volunteer. A compliance rate ≥80% indicates a good compliance. To improve compliance, a WeChat group is established for the cohort of volunteers, and the study personnel can post messages via the WeChat group, reminding volunteers to take the samples as scheduled and complete the defecation diary (Appendix 2). In addition, each volunteer can receive 300 RMB as a reward once they complete the follow-up.

Volunteers can withdraw from the trial for the following reasons [1]: Persistent adverse reactions (cannot be resolved after taking certain measures) possibly involving the four kinds of AEs listed in “Safety evaluation part” [2,39] Volunteers can autonomously withdraw from the study at any time for any reason.

### 2.13 Sample size

The trial obeys the principle of a superiority design. Combined pilot cases observation and experts’ opinion [35,36], it is estimated that the primary outcome, diarrhoea severity score on day 28, can differ by 3 points between the probiotics or placebo groups, with a standard deviation of 6.8. Given an *α* = 0.05 and a *β* = 0.20, the sample size is calculated to be at least 81 per group. After considering the drop-out rate (20% or lower), the sample size should be 97 or more per group. The final sample size is determined to be 100 per group with a total of 200 cases.

### 2.14 Data collection and monitoring

An independent data management team (DMT) is formed to periodically assess the study quality and safety. Volunteers are required to complete the defecation diary (Appendix 2) daily online and the DASS-21 (Appendix 3) online as scheduled in Table 1, both of which are promptly downloaded and collected by the data analysts. The data of demographic profiles, defecation diary (Appendix 2) and DASS-21 (Appendix 3) completed online by volunteers can downloaded as excel spreadsheets for further analysis. For gut microbiome and metabolomics test, volunteers have to prepare stool samples as the instructions of test kit at home, at day 0 (observing period of pre-administrating), 28 (observing period of administrating) and 42 (observing period of pro-administrating), all of which are timely collected, separately stored and sent for detection at −80 °C in a special stool storage kit from Guangdong longsee company (www.longseemed.com). The data of gut microbiome and metabolomics detected from stool samples will detected by Key Laboratory of Dairy Biotechnology and Engineering, Ministry of Education.

All the data will be submitted to the DMT for managing and monitoring. The DMT will promptly check the data and urge the data collectors to resolve it when uncertainty about the data occurs. All records that contain names, ID Number, or other personal identifiers (e.g., informed consent forms), are stored separately from study records. The database will be password protected by the DMT. The team also has the authority to carry out an interim analysis or terminate the next-step study according to the following situations [1]: if the probiotics does not present effect on gut microbiome, then the study will not further conduct the detections of faecal metabolome [3]; if any severe adverse events related to probiotics occurs, DMT will suggest terminating the study.

### 2.15 Statistical analysis

The following four datasets will be used for the analysis [1]: the data of all volunteers who completed the follow-up and [2] the data of three subgroups divided according to compliance (≥80%, 60–80%, and <60%), which will be analysed to validate the robustness of the analysis results. The following analysis methods will be used to analyse each dataset.

First, to analyse the inter-group differences in each outcome measure, Z test (as the sample size is quite more than 10, using Z test instead of Wilcoxon signed-rank sum test) will be used for the outcome measures, including the diarrhoea severity score, weekly mean frequency of defecation, weekly mean stool appearance score, weekly mean stool urgency score, and emotional state score, at each observation point. In the analyses, the *P*-value indicating statistical significance will be set at 0.05. More detailed information about how to analyse and present inter-group differences is clearly provided in Table 2.

**Table 2:** The format to analyse and present the inter-group differences between probiotic and placebo groups at each observation point.

Groups	P5	P25	Median	P75	P95	Z value	*P*
*L. plantarum* p9							
Placebo							

Note: The data applied here includes the diarrhoea severity score, weekly mean frequency of defecation, weekly mean stool appearance score, weekly mean stool urgency score, and emotional state score; P5, P25, P75, P95 means the 5th, 25th, 75th, 95th percentile, respectively; Z = |T-n1*(N+1)/2–0.5|/SQRT [n1*n2(N+1)/12], n1 refers to the smaller sample size between the two compared groups, while n2 refers to the sample size of the other group; N = n1+n2; T is calculated using method of Wilcoxon signed-rank sum test.

Then, to identify the intra-group changing trends with the consecutive phases, namely, pre-, during and post-probiotic administrating, the *Kruskal-Wallis* rank sum test will be used for multiple comparisons of the diarrhoea severity score, weekly mean frequency of defecation, weekly mean stool appearance score, weekly mean stool urgency score, and emotional state score for each group, i.e., probiotic group or placebo group. In the analyses, the *P*-value indicating statistical significance will be adjusted to be 0.05/[k(k-1)/2], where k is the number of sample groups in the comparison. More detailed information about how to analyse and present inter-group differences is clearly provided in Table 3.

**Table 3:** The format to analyse and present the intra-group differences among the four observation points in a probiotic or placebo group.

Groups	P5	P25	Median	P75	P95	H value	*P*
Day 0							
Day 14							
Day 28							
Day 42							

Note: The data here includes the diarrhoea severity score, weekly mean frequency of defecation, weekly mean stool appearance score, weekly mean stool urgency score, and emotional state score; P5, P25, P75, P95 means the 5th, 25th, 75th, 95th percentile, respectively; H is calculated using method of *Kruskal-Wallis* rank sum test.

In addition, the statistical analyses of metagenomics and metabolomics will be performed using the R software (v.4.0.2) and Adobe Illustrator. PCA and PLS-DA analysis will be performed and visualized using the R package vegan, ggplot and ggpubr, and the adonis *P*-value will be generated based on 999 permutations. *t*-test, *Wilcoxon* test and *Kruskal-Wallis* test will be used to evaluate differences in various variables between and within groups; *P* values will be corrected for multiple testing using the Benjamini-Hochberg procedure. Meanwhile, Pearson and spearman correlations will be used to analyse the correlations between different indicators in clinical, metagenomics and metabolomics.

## 3 Discussion

Chronic diarrhoea can disturb the patient's quality of life, work performance and wellbeing, increase patient's expenses [10], and leads to economical loss because of work-loss [11]. Probiotics may offer a way for the problems [[12], [13], [14], [15], [16], [17],^31^]. However, clinical trials pin-pointing the effectiveness of probiotics for chronic diarrhoea are currently limited. In this specific context, we design the study protocol. This is the first study protocol designed to assess whether the probiotic can improve the symptoms of patients with chronic diarrhoea (previous studies using nonhuman primates or not only focus on chronic diarrhoea).

Some special steps are taken to guarantee the eligibility of volunteers, the feasibility of study, and interpretability of results, including completing diary online daily (for recording the times, appearance and urgency feeling of defecation), face-to-face consultation with the clinical specialist (confirming the realness of information provided online by volunteers), and stool test (excluding intestinal organic lesion) in the 14 days screening period. The study intervention, *L. plantarum* p9, belongs to *Lactobacillus* species, which is one of the most studied probiotics [41,42] and reported to be beneficial in reducing the risk of developing *Clostridium difficile*-associated diarrhoea [17,43] and the overall risk of diarrhoea lasting four or more days by 59% and the average duration thereof by 25 h [44].

Multidimensional outcomes are examined to produce a chain of evidence from causal improvements in metagenomic and metabolomic data to clinical symptoms and then to the emotional state. Chronic diarrhoea can disturb the patient's quality of life, work performance and wellbeing [10]; thereby, the relevant outcomes are chosen, including the diarrhoea severity score [33,34], weekly mean frequency of defecation, weekly mean stool appearance score [33,37], weekly mean stool urgency score [34], emotional state score [38] according to corresponding references [33,34,37,38]. The diarrhoea severity score is a comprehensive indicator calculated from weekly mean frequency of defecation, weekly mean stool appearance score, and weekly mean stool urgency score as described in “Primary outcome part”, so it is more reasonable to be the primary outcome than the left. Furthermore, both of inter- and intra-group differences will be analysed. In our opinion, the results of analysis are appropriate to present the effect of *L. plantarum* P9 on defecation and quality of life in individuals with chronic diarrhoea. Moreover, the study will try to explore the possible action modes of probiotics to improve chronic diarrhoea, from the aspects of gut microbiome and faecal metabolome. Gut microbiome has been found to be altered in chronic diarrhoea patients and may be a cause of chronic diarrhoea [12]. Probiotics are live organisms that when administered in adequate amounts confer health benefits to the host and improve the gut microbial balance [[13], [14], [15], [16], [17],^26^]. The detection of gut microbiome and faecal metabolome would provide an explanation for the efficacy of *L. plantarum* P9 on chronic diarrhoea. Thus, the set of multidimensional outcomes will form a chain of evidence.

A limitation is the way of follow up in this study (completing diary online daily), which may lead to bias about the effectiveness of probiotics. To overcome the limitation, a WeChat group is established for the cohort of volunteers, so the researcher can timely post messages via the WeChat group, keeping a good compliance; and an DMT is formed to promptly assess the study quality and safety. during the assessment, once an uncertainty about the data arises, the DMT can urge the data collectors to resolve it. The confounding factors are also prohibited. In such a way, the bias would be minimized as far as possible.

Collectively, the study protocol will provide high-quality evidence for the use of probiotics as a diarrhoea agent when it is strictly conducted out, providing evidence regarding whether and to what extent *L. plantarum* p9 can improve the defecation and well-being of individuals with chronic diarrhoea. If the study findings are as expected, *L. plantarum* p9, taken as probiotics, may become an optimal nonmedical intervention option for patients with chronic diarrhoea.

## 4 Trial status

This study started recruiting patients on October 1, 2020. To date, 174 patients have been enrolled. The enrolment is expected to be completed by October 31, 2022, and all follow-ups are expected to be completed by December 31, 2022. The protocol is 2.0 version, and the date of edition was September 10, 2020.

## Consent for publication

Not applicable.

## Ethics approval and consent to participate

This study was registered at Chinese Clinical Trial Registry (ChiCTR) (NO. ChiCTR2000038410) on November 22, 2020 (Appendix 4), and approved on November 14, 2020, by the Ethics Committee of the first affiliated hospital of Nanchang university (NO. IIT [2020] Clinical Ethics Review No. 002 (Appendix 5). All participants will sign informed consent and consent to initial assessment and participate in this study (Appendix 6).

## Availability of data and materials

Data sharing is not applicable to this trial as no database is generated or analysed for the current study. And when the study completed, the study results will be released to the public, volunteers, and the general medical community via publishing journal article, with all the related data being available. The process of publication will be independent from the funder and sponsor.

## Funding

The trail is supported by the National Natural Science Foundation of China (31720103911) to Heping Zhang (Appendix 7). *L. plantarum* P9 probiotics and placebo will be manufactured by Jiangzhong Pharmaceutical Company Limited (Nanchang, China). Jiangzhong Pharmaceutical Company Limited also is a sponsor and can be contacted to Jianhua Wan at email: wanjianhua@crjz.com. The funders play no role in the design of study, data collection and analysis, or preparation of the manuscript.

## Authors’ contributions

WJL, NHL and HPZ conceived and designed this research and supervised the research team. TLX, YML, YX, and LJZ are the main implementers of the study and WFZ, QPX, NY and KXZ help with implementation. TLX drafted the manuscripts and participated in the design of the study. YML and XZ assisted in designing the study and drafting the manuscript. The authorship is based on the authors' contributions. All authors have read and agreed to the final version of the manuscript.

## Declaration of competing interest

The authors declare that they have no competing interests. And the study has not received funding/assistance from a commercial organization.
