# Supplementary material for: Effect of Lactobacillus plantarum P9 on defecation, quality of life and gut microbiome in individuals with chronic diarrhoea: Protocol for a randomized, double-blind, placebo-controlled clinical trial
Source: Contemp Clin Trials Commun. 2023 Feb 1;32:101085. doi: 10.1016/j.conctc.2023.101085 (PMC9970898; doi:10.1016/j.conctc.2023.101085)
Supplement: Multimedia component 1 [file mmc1.docx]

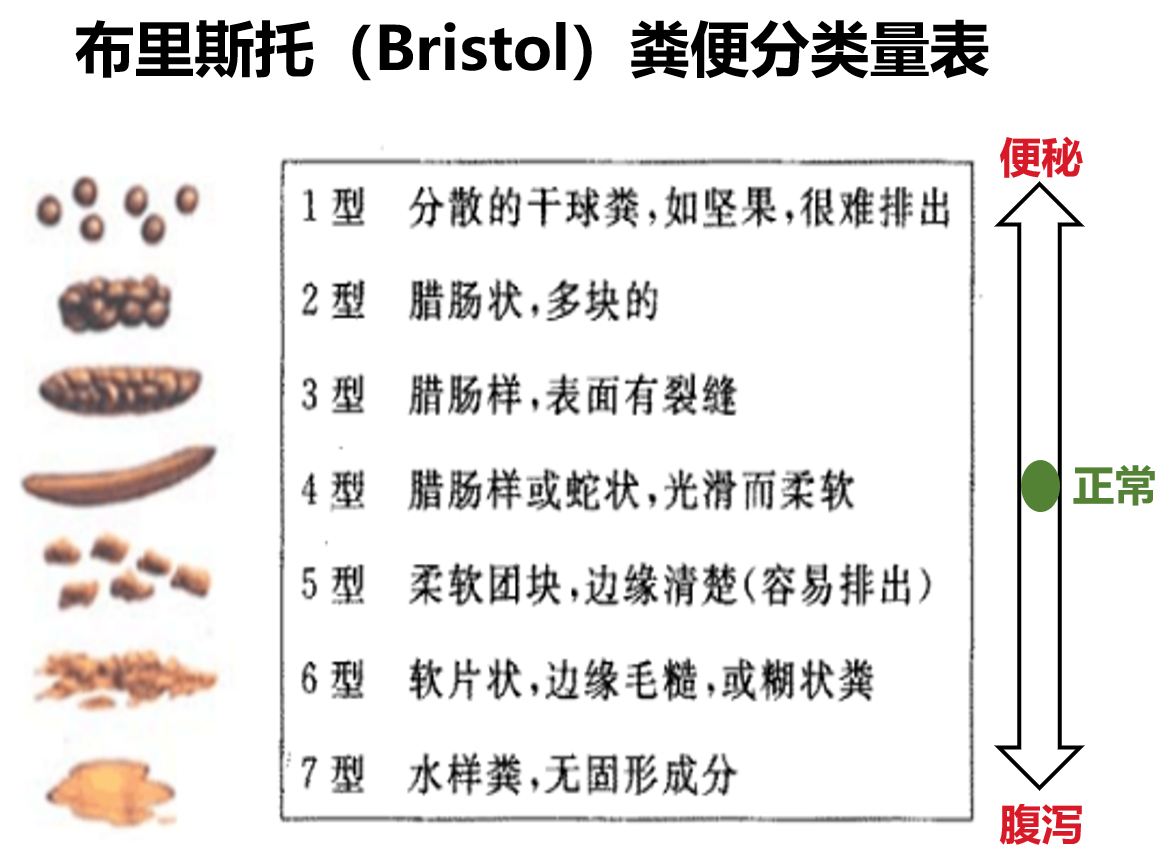


**Type 5: Somewhat loose: soft blobs with clear edges (easy to pass)**

**Type 1: Separate hard lumps, like nuts (difficult to pass)**

**Diarrhoea**

**Type 7: Watery: no solid pieces**

**Type 6: Runny: fluffy pieces with ragged edges, or a mushy stool**

**Type 4: Normal consistency: sausage- or snake-like; smooth and soft**

**Type 3: Sausage-like, with cracks on the surface**

**Type 2: Sausage-shaped, lumpy**

**Constipation**

**Normal**

**Appendix 1** **Bristol Stool Form Scale** [1]

1. Blake MR, Raker JM, Whelan K. Validity and reliability of the Bristol Stool Form Scale in healthy adults and patients with diarrhoea-predominant irritable bowel syndrome. Aliment Pharmacol Ther. 2016; 44:693-703.
