# Supplementary material for: Effect of Lactobacillus plantarum P9 on defecation, quality of life and gut microbiome in individuals with chronic diarrhoea: Protocol for a randomized, double-blind, placebo-controlled clinical trial
Source: Contemp Clin Trials Commun. 2023 Feb 1;32:101085. doi: 10.1016/j.conctc.2023.101085 (PMC9970898; doi:10.1016/j.conctc.2023.101085)
Supplement: Multimedia component 2 [file mmc2.docx]

| **1. Date: __ __ (day)__ __ (month) 20__ __(year) Volunteer ID:____________** | | |
| --- | --- | --- |
| **Defecation: □ Yes □ No** | Frequency of defecation today: ______ | |
| **The appearance and urgency in each time of defecation** | | |
| **Time** | Stool type (appearance) | Stool urgency |
| **1^st^** | Type:______ | points |
| **2^nd^** | Type:______ | points |
| **3^rd^** | Type:______ | points |
| **4^th^** | Type:______ | points |
| **5^th^** | Type:______ | points |
| **6^th^** | Type:______ | points |
| **7^th^** | Type:______ | points |
| **2. Special situations related to today’s defecation** | | |
| **1) Is today’s abnormal defecation related to changes in dietary habits (such as eating spicy or oily foods or drinking)?**  **□ Yes □ No** | | |
| **2) Did you take antibiotics today? □ Yes □ No**  **If “yes”, please provide the details, including the name of the drug, daily dose, dosage unit, route of administration (oral, intravenous, or intramuscular), and reason for taking antibiotics.** | | |
| **3) Is there any other situation that you think may have affected your defecation? □ Yes □ No**  **If “yes”, please provide the details.** | | |
| **Notes:** | | |

**Appendix-2: Defecation diary**
