# Supplementary material for: Effect of Lactobacillus plantarum P9 on defecation, quality of life and gut microbiome in individuals with chronic diarrhoea: Protocol for a randomized, double-blind, placebo-controlled clinical trial
Source: Contemp Clin Trials Commun. 2023 Feb 1;32:101085. doi: 10.1016/j.conctc.2023.101085 (PMC9970898; doi:10.1016/j.conctc.2023.101085)
Supplement: Multimedia component 3 [file mmc3.docx]

**Depression Anxiety Stress Scale 21** [1]

Please read each statement and select the response which best describes how much the statement applied to you over the past week. Please try to answer every question. There are no right or wrong answers.

The rating scale is:

0 — Did not apply to me at all

1 — Applied to me some degree, or some of the time

2 — Applied to me a considerable degree, or a good part of time

3 — Applied to me very much, or most of the time

| 1.  I found it hard to wind down. | 0 | 1 | 2 |
| --- | --- | --- | --- |
| 2.  I was aware of dryness of my mouth. | 0 | 1 | 2 |
| 3.  I couldn’t seem to experience any positive feeling at all. | 0 | 1 | 2 |
| 4.  I experienced breathing difficulty. | 0 | 1 | 2 |
| 5.  I found it difficult to work up the initiative to do things. | 0 | 1 | 2 |
| 6.  I tended to over-react to situations. | 0 | 1 | 2 |
| 7.  I experienced trembling (e.g., in the hands). | 0 | 1 | 2 |
| 8.  I felt that I was using a lot of nervous energy. | 0 | 1 | 2 |
| 9.  I was worried about situations in which I might panic and make a fool of myself. | 0 | 1 | 2 |
| 10.  I felt that I had nothing to look forward to. | 0 | 1 | 2 |
| 11.  I found myself getting agitated | 0 | 1 | 2 |
| 12.  I found it difficult to relax. | 0 | 1 | 2 |
| 13.  I felt downhearted and blue. | 0 | 1 | 2 |
| 14.  I was intolerant of anything that kept me from getting on with what I was doing. | 0 | 1 | 2 |
| 15.  I felt I was close to panic. | 0 | 1 | 2 |
| 16.  I was unable to become enthusiastic about anything. | 0 | 1 | 2 |
| 17.  I felt that I wasn’t worth much as a person. | 0 | 1 | 2 |
| 18.  I felt I was rather touchy. | 0 | 1 | 2 |
| 19. I was aware of the action of my heart in the absence of physical exertion. | 0 | 1 | 2 |
| 20.  I felt scared without any good reason. | 0 | 1 | 2 |
| 21.  I felt that life was meaningless. | 0 | 1 | 2 |

1. Lovibond PF, Lovibond SH. The structure of negative emotional states: comparison of the Depression Anxiety Stress Scales (DASS) with the Beck Depression and Anxiety Inventories. Behav Res Ther. 1995; 33:335-43.
