# Supplementary material for: Effect of Lactobacillus plantarum P9 on defecation, quality of life and gut microbiome in individuals with chronic diarrhoea: Protocol for a randomized, double-blind, placebo-controlled clinical trial
Source: Contemp Clin Trials Commun. 2023 Feb 1;32:101085. doi: 10.1016/j.conctc.2023.101085 (PMC9970898; doi:10.1016/j.conctc.2023.101085)
Supplement: Multimedia component 4 [file mmc4.docx]

| **Data category** | **Information** |
| --- | --- |
| Primary registry and trial identifying number | https://www.chictr.org.cn/index.aspx ChiCTR2000038410 |
| Date of registration in primary registry | 22 September, 2020 |
| Source(s) of monetary or material support | The trail was supported by the National Natural Science Foundation of China (31720103911) and Jiangzhong Pharmaceutical Company Limited to Heping Zhang. |
| Primary sponsor | Jiangzhong Pharmaceutical Co., Ltd. |
| Public title | Effect of *Lactobacillus plantarum* P9 on defecation, quality of life and gut microbiome in patients with chronic diarrhea |
| Scientific title | *Effect of Lactobacillus plantarum P9 Probiotics on defecation, quality of life and gut microbiome in individuals with chronic diarrhea: a randomized, double-blind, placebo-controlled clinical trial* |
| Countries of recruitment | China |
| Health condition(s) or problem(s) studied | Chronic diarrhea；Probiotics |
| Intervention(s) | ***Probiotics group:*** *Volunteers will take Lactobacillus plantarum P9 powder directly or with warm water (below 40℃) on a full stomach, 1 pack (100 billion CFU) per day.*  ***Placebo group：****Volunteers will take the placebo with the same manner as the probiotics group. The placebo contains no probiotics, comprises maltodextrin (60%), orange powder (20%), and maltitol (20%) and has the same appearance, packaging, and taste as the Lactobacillus plantarum P9 powder.* |
| Key inclusion and exclusion criteria | **Inclusion criteria:** Eligible volunteers should have diarrhea symptoms for at least 6 months before enrolment, with loose or watery stool (looked like Bristol type 5, 6, or 7) at least 25% of the times of defecation within the past 3 months. The volunteers involved in this study will be patients with chronic diarrhea aged 18-65 years. For patients aged from 18 (exclusive) to 50 (inclusive) years, the result of stool test (including occult blood) conducted during the screening period is normal or is abnormal but is judged by the investigators as clinically irrelevant. For patients aged from 50 (exclusive) to 65 (inclusive) years, the result of colonoscopy performed at a tertiary or higher-level hospital within the past 6 months is normal or is abnormal but must be judged by the investigators as clinically irrelevant. Of course, the volunteers should be willing to participate in this trial and sign the informed consent form. |
|  | **Exclusion criteria：**Volunteers with any of the following situations will be excluded.  (1) Personal or family history of colon cancer, celiac disease, or inflammatory bowel disease.  (2) Intestinal organic diseases confirmed with previous colonoscopy.  (3) Plans to become pregnant or father a child in the next 3 months, or pregnancy or breastfeeding in women.  (4) Allergies to samples or ingredients.  (5) Use of antibiotics or probiotics within the past two weeks.  (6) Use of antianxiety, antidepressant, or other psychotropic drugs within the past month.  (7) Need for long-term use of medications for diarrhoea.  (8) History of severe diseases, such as myocardial infarction, cerebral infarction, and malignant tumour, judged by the investigators as disqualifying conditions.  (9) Major mental illnesses, inability to control one’s actions, or inability to cooperate.  (10) Illiteracy, inability to understand the informed consent form, or inability to independently sign the informed consent form. |
| Study type | Interventional |
|  | Allocation: randomized intervention model. Parallel assignment masking: double blind (subject, investigator, caregiver, outcomes assessor) |
|  | Primary purpose: prevention |
| Date of first enrolment | October 1, 2020 |
| Target sample size | 200 |
| Recruitment status | Recruiting |
| Primary outcome(s) | The primary outcome is the diarrhea severity score assessed by the Gastrointestinal Symptom Rating Scale (GSRS) |
| Key secondary outcomes | The secondary outcome measures include weekly mean frequency of defecation, weekly mean stool appearance score, weekly mean stool urgency score, emotional state score, gut microbiome and fecal metabolome. |
