# Supplementary material for: Effect of Lactobacillus plantarum P9 on defecation, quality of life and gut microbiome in individuals with chronic diarrhoea: Protocol for a randomized, double-blind, placebo-controlled clinical trial
Source: Contemp Clin Trials Commun. 2023 Feb 1;32:101085. doi: 10.1016/j.conctc.2023.101085 (PMC9970898; doi:10.1016/j.conctc.2023.101085)
Supplement: Multimedia component 5 [file mmc5.docx]

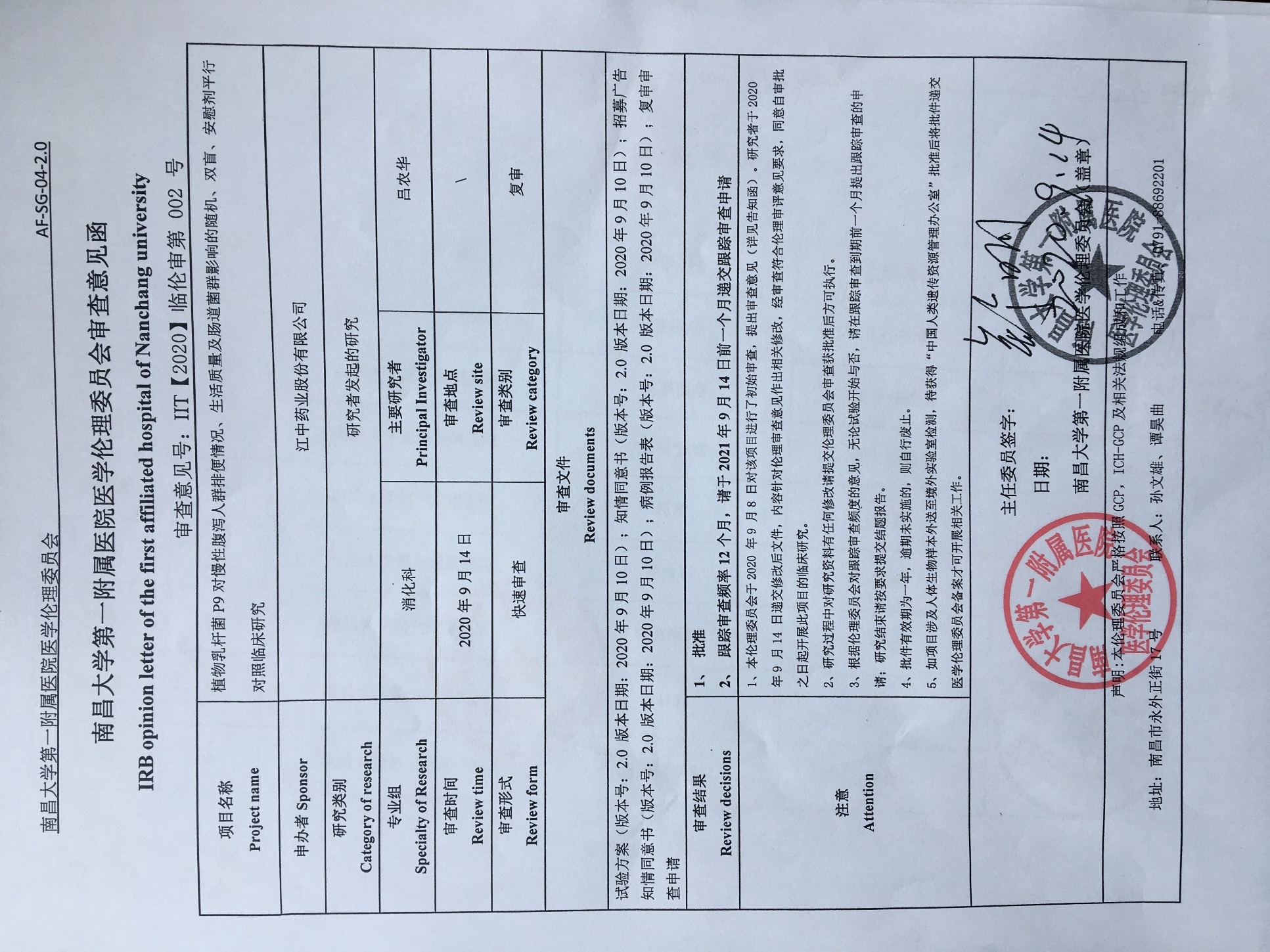


IRB opinion letter of the first affiliated hospital of Nanchang university

Review opinion number: IIT [2020] Clinical Ethics Review No. 002

| Project name | Effect of *Lactobacillus plantarum* P9 on defecation, quality of life and gut microbiome in individuals with chronic diarrhea: a randomized, double-blind, placebo-controlled clinical trial | | |
| --- | --- | --- | --- |
| Sponsor | Jiangzhong Pharmaceutical Co., Ltd. | | |
| Category of research | investigator-initiated clinical trial | | |
| Specialty of Research | Gastroenterology | Principal Investigator | Nonghua Lu |
| Review time | September 14, 2020 | Review site | \ |
| Review form | Quick review | Review category | Reexamination |
| Review documents | | | |
| clinical trial protocol（Version no.:2.0 Version Date: 10 September 2020)；Informed consent (Version no.: 2.0 Version Date: 10 September 2020); Recruitment advertisement (Version no.: 2.0 Version Date: 10 September 2020); Case Report Form (Version no.: 2.0 Version Date: 10 September 2020); Application for review | | | |
| Review decisions | 1. Approve 2. The frequency of follow-up review is 12 months. Please submit the application for follow-up review one month before September 14, 2021 | | |
| Attention | 1. The ethics committee conducted an initial review of the project on September 8, 2020, and put forward some corrections (see the notification letter for details). The researcher submitted the revised documents on September 14, 2020, and the content was revised in response to the ethical review opinions. Reexamination found that the revised documents met the requirements of the ethical review opinion, thus, it was agreed to start the clinical research of this project from the date of approval. 2. Any change to the research program during the study should be submitted to the ethics committee for review and need approval before implementation. 3. According to the suggestion of the ethics committee on the frequency of follow-up review, no matter whether the trial is started or not, please submit an application for follow-up review one month before the expiration of the follow-up review. After completing the research, please submit a final report as required. 4. The approval document is valid for one year, if it is not implemented within the time limit, it will be automatically annulled. 5. If the project involves sending biological samples of human bodies to overseas laboratories for testing, it is essential to submit an application to the Medical Ethics Committee for record, and obtain the approval of the “China Human Genetic Resources Management Office” before the relevant work can be carried out. | | |
| Chairman's signature：Ming Shu  Date：September 14, 2020  Medical Ethics Committee of the First Affiliated Hospital of Nanchang University（[seal](C:/Users/13424/AppData/Local/youdao/dict/Application/8.9.6.0/resultui/html/index.html#/javascript:;)）： | | | |
| Statement: The ethics committee is found and conduct work in strict accordance with GCP ICH-GCP and related regulations  Address: No. 17 Yongwaizheng Street, Nanchang City Contact: Sun Wenxiong, Tan Haoqu Tel & Fax：0791-88692201 | | | |


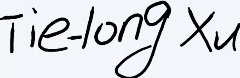


Signature:
