# Supplementary material for: Effect of Lactobacillus plantarum P9 on defecation, quality of life and gut microbiome in individuals with chronic diarrhoea: Protocol for a randomized, double-blind, placebo-controlled clinical trial
Source: Contemp Clin Trials Commun. 2023 Feb 1;32:101085. doi: 10.1016/j.conctc.2023.101085 (PMC9970898; doi:10.1016/j.conctc.2023.101085)
Supplement: Multimedia component 6 [file mmc6.docx]

Effect of *Lactobacillus plantarum* P9 on defecation, quality of life and gut microbiome in

individuals with chronic diarrhea: a randomized, double-blind, placebo-controlled clinical

trial

**Patient informed consent**

Dear patient, you will be invited to participate in a clinical study. This study is jointly sponsored by the First Affiliated Hospital of Nanchang University, Inner Mongolia Agricultural University, and Jianzhong Pharmaceutical Co., Ltd. The study is a randomized, double-blind, placebo, parallel-controlled clinical study of the effects of Lactobacillus plantarum P9 on defecation, quality of life in people with chronic diarrhea.

We hereby explain to you the following contents：

**1. Background and objective**

Diarrhea is defined as the situation that the stool weight is more than 200 gram per 24 hours containing more than 200 ml fluid per 24 hours, or the times of defecating loose stools are greater than 3 within 24 hours. An individual can be diagnosed as chronic diarrhea when diarrhea lasts more than 4 weeks. The prevalence of chronic diarrhea is estimated to be 3-5% among general population. However, it may be somewhat more common in the elderly (aged more than 60) than in the general population, with the prevalence reaching to 9.6-14.2%. Chronic diarrhea poses some specific challenges. It has many differential causes that confusing the diagnosis. It may also disturb the patient’s quality of life, work performance and well-being as well as increase their medical expenses, and the chronic diarrhea-related economical loss just from work-loss is estimated to be approximate $350,000,000 annually.

Probiotics may offer a way for the problems [12-17, 29]. However, clinical trials pin-pointing the effectiveness of probiotics for chronic diarrhea are currently limited. In this specific context, we design and conduct the study.

**2. Introduction of *Lactobacillus plantarum* P9**

“Lihuo Probiotic Solid Beverages” is a probiotic product that has been marketed by Jiangzhong Pharmaceutical Co., Ltd.，which is composed of *Bifidobacterium lactis* V9, *Lactobacillus casei* Zhang, and *Lactobacillus plantarum* P9. *Lactobacillus plantarum* P9 is one of the three strains of probiotic solid beverages that are already on the market. *Lactobacillus plantarum* P9 is a probiotic strain isolated from natural fermented sour porridge in Bayannaoer, Inner Mongolia. Previous studies have shown that *Lactobacillus plantarum* P9 can regulate the intestinal flora, increase beneficial bacteria, and decrease harmful bacteria.

**3. Criteria for participating the trials**

Trained study implementers and clinical specialists discuss the criteria of participating the trials with you about the information provided in the video and information sheets. Please provide related information, so as to judge whether you are eligible to participate the trial：

Inclusion criteria

Eligible volunteers should have diarrhea symptoms for at least 6 months before enrolment, with loose or watery stool (looked like Bristol type 5, 6, or 7, showed as Appendix 1) at least 25% of the times of defecation within the past 3 months. The volunteers involved in this study will be patients with chronic diarrhea aged 18-65 years. For patients aged from 18 (exclusive) to 50 (inclusive) years, the result of stool test (including occult blood) conducted during the screening period is normal or is abnormal but is judged by the investigators as clinically irrelevant. For patients aged from 50 (exclusive) to 65 (inclusive) years, the result of colonoscopy performed at a tertiary or higher-level hospital within the past 6 months is normal or is abnormal but must be judged by the investigators as clinically irrelevant. Of course, the volunteers should be willing to participate in this trial and sign the informed consent form.

Exclusion criteria

Volunteers with any of the following situations will be excluded.

(1) Personal or family history of colon cancer, celiac disease, or inflammatory bowel disease.

(2) Intestinal organic diseases confirmed with previous colonoscopy.

(3) Plans to become pregnant or father a child in the next 3 months, or pregnancy or breastfeeding in women.

(4) Allergies to samples or ingredients.

(5) Use of antibiotics or probiotics within the past two weeks.

(6) Use of antianxiety, antidepressant, or other psychotropic drugs within the past month.

(7) Need for long-term use of medications for diarrhoea.

(8) History of severe diseases, such as myocardial infarction, cerebral infarction, and malignant tumour, judged by the investigators as disqualifying conditions.

(9) Major mental illnesses, inability to control one’s actions, or inability to cooperate.

(10) Illiteracy, inability to understand the informed consent form, or inability to independently sign the informed consent form.

**4. Research groups and interventions**


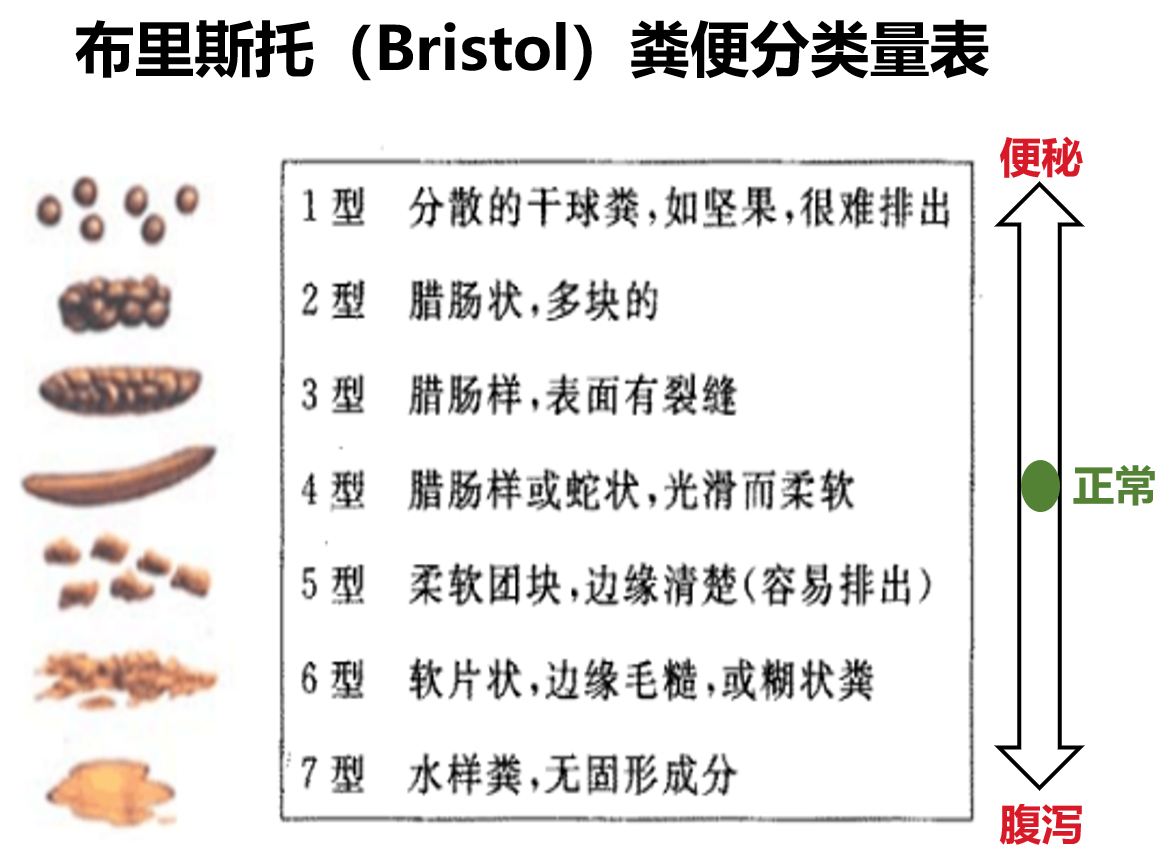


**Type 5: Soft blobs with clear edges (easy to pass)**

**Type 1: Separate hard lumps, like nuts (difficult to pass)**

**Diarrhoea**

**Type 7: Watery; no solid pieces**

**Type 6: Fluffy pieces with ragged edges, or a mushy stool**

**Type 4: Sausage- or snake-like; smooth and soft**

**Type 3: Sausage-like, with cracks on the surface**

**Type 2: Sausage-shaped, lumpy**

**Constipation**

**Normal**

**Appendix 1** **Bristol Stool Form Scale**

If you agree to participate in this research, please sign this informed consent form. The study will include three phases, including a period of screening (an observation period of pre-administrating), an observation period of administrating, and an observation period of post-administrating. The interventions in each phase will be as follows:

(1) Period of screening (observation period of pre-administrating) (days -14 to 0): After signing an informed consent form, you will undergo a 14-day screening period when you will not be allowed to take any medicines or health products to improve their diarrhea symptoms. Additionally, you will be asked to collect one stool sample and complete an online daily diary (Figure 2). At the end of the screening period, the diary and stool exam results will be reviewed and used to judge whether you are an eligible volunteer according to the inclusion and exclusion criteria. The following steps will be the formal intervention and follow-ups.

A random sequence will be generated by the computer and used to randomly assign you to a probiotic group or a placebo group.

(2) Observation period of administrating (days 0 to 28):

1) Probiotics group: Volunteers will take *Lactobacillus plantarum* P9 powder directly or with warm water (below 40℃) on a full stomach, 1 pack (100 billion CFU) per day; if antibiotics must be taken, probiotics should be taken 2 hours later.

2) Placebo group: Volunteers will take the placebo with the same manner as the probiotics group. The placebo contains no probiotics, comprises maltodextrin (60%), orange powder (20%), and maltitol (20%) and has the same appearance, packaging, and taste as the Lactobacillus plantarum P9 powder.

Both probiotics and placebo will be stored in a cool, dry place away from direct sunlight.

(3) Observation period of post-administrating (days 29 to 42): No probiotics or placebo will be taken during the study. All remaining probiotics or placebo possibly untaken and empty packages which are used to hold probiotics or placebo will be collected at the end of this period, to monitor compliance.

During each treatment cycle, you need to cooperate with your doctor’s evaluation. Your doctor will periodically conduct various evaluation on you in accordance with the regulations of the research plan to evaluate the efficacy and safety of the sample after you receive the treatment.

Additionally, one stool sample for gut microbiome (essential), one for metabolomics test (essential) and one whole blood sample for microRNA detection (optional) will be taken from each volunteer at day 0 (observing period of pre-administrating), 28 (observing period of administrating) and 42 (observing period of pro-administrating).

**5. Possible benefits of participating in the study**

Your medical condition may be improved by participating in this study. Expected improvements may include diarrhea severity, number of bowel movements, gut flora, etc., but we cannot guarantee that you will benefit from this study.

You will get probiotics free for 28 days. In addition, each volunteer will receive a reward of 300 RMB once they complete the follow-up.

**6. Participate in the study/withdraw from the study/terminate the study**

Whether to participate in the study is entirely up to your volition. You can refuse to participate in this study, or you can withdraw from this study at any time during the study process. This will not affect the relationship between you and the doctor, and will not cause the loss of your medical or other benefits.

**7. Alternative treatment options**

In addition to participating in this study, you can choose to receive conventional treatments provided by doctors in the clinic, such as adsorbents, anti-motility agents, etc. for treatment. You can discuss other treatments with your doctor.

**8. Confidentiality in the study**

If you agree to participate in this study, your medical records will be reviewed by the executor and supervisor of the study. All your information collected during the research will be kept strictly confidential, and only your contact information will be listed in a form containing identifiable information. We will save the form in a secure database, and we are ready to contact you by phone when we need it in the future. However, during data analysis, all your information will be de-marked, and no personal information will be disclosed in future publications or other articles published to the public.

**9. Risks and discomforts in the study**

After taking probiotics, it may cause a gurgling sound in the stomach and produce a little more gas. Some studies have shown that people who are intolerant to taking probiotics can cause diarrhea. This is considered a "Herxcel reaction". This reaction usually takes a short time, and most people disappear within a few days to 2 weeks.

Any scientific research has risks, discomforts and inconveniences, so you should fully consider it before agreeing to participate in any clinical research.

**10. experimental expense**

During the clinical study period, the sponsor will provide the probiotic product lactobacillus plantarum P9 and placebo free of charge until the end of the study and provide the examination fees stipulated in the study protocol. During the study period, if there are serious adverse reactions related to the experimental drugs, Jiangzhong Pharmaceutical Co., Ltd. will provide reimbursement of the corresponding treatment costs and corresponding economic compensation for the damage related to the study in accordance with the relevant laws and regulations of China.

Any other medical conditions that you may have at the same time will not be reimbursed.

**Signature page**

**If you or your family/guardian agree to participate in the study, please read the relevant statement in detail and sign.**

I have been informed of the purpose, method, and possible risks, discomforts, and related benefits of this trial.

I am sure that I have spent enough time to read and understand the above content, the doctor has explained to me the medical terms used in it, and I have given satisfactory answers to all the questions raised by the research. I understand that I can voluntarily withdraw from this study at any time without affecting the future doctor-patient relationship and treatment. I know that if you have any questions during the trial, you should contact the doctor in charge in time.

I voluntarily participate in this trial and become a subject of this trial.

Signature of volunteer:

Date of signature:

I have truthfully informed the subject (or designated agent) of the purpose, content, benefits, and possible adverse reactions of this research. I have asked the subject if they have any questions about this research and have tried my best to explain it.

Signature of researcher:

Date of signature:
