# Supplementary material for: Effect of Lactobacillus plantarum P9 on defecation, quality of life and gut microbiome in individuals with chronic diarrhoea: Protocol for a randomized, double-blind, placebo-controlled clinical trial
Source: Contemp Clin Trials Commun. 2023 Feb 1;32:101085. doi: 10.1016/j.conctc.2023.101085 (PMC9970898; doi:10.1016/j.conctc.2023.101085)
Supplement: Multimedia component 7 [file mmc7.docx]

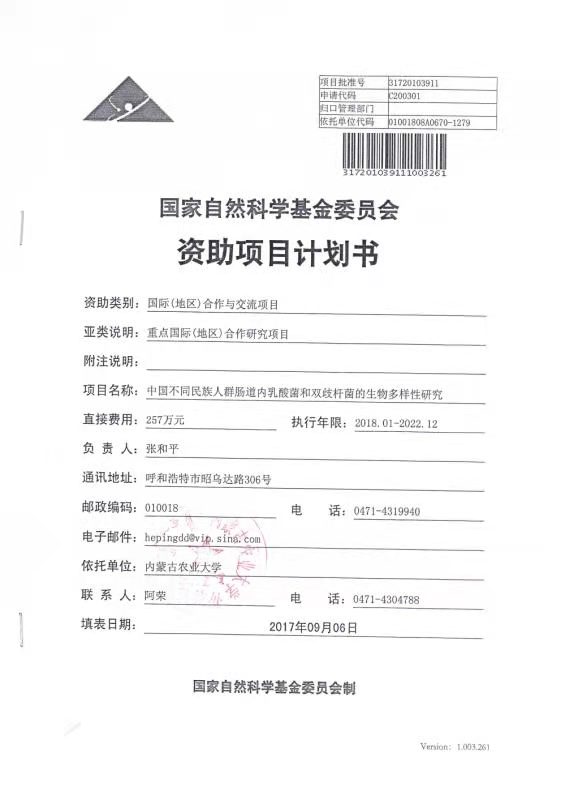


Project approval number: 31720103911

Application code: C200301

Central Management Department:

Relying unit code: 01001808A0670-1279

National Natural Science Foundation of China

Funding project plan

Funding category: International (regional) cooperation and exchange projects

Sub-category description: Key international (regional) cooperative research projects

Note description:

Project name: Study on Biodiversity of *Lactobacillus* and *Bifidobacterium* in the Intestines of Different Ethnic Groups in China

Direct cost: ￥2.57 million Execution period: January 2018 to December 2022

Person in charge: Heping Zhang

Mailing address: No. 306, Zhaowuda Road, Hohhot

Postal Code: 010018 Phone: 0471-439940

Email: hepingdd@vip.sina.com

Supporting unit: Inner Mongolia Agricultural University

Contact: A Rong Phone: 0471-4304788

Date of completion: September 06, 2017

National Natural Science Foundation of China


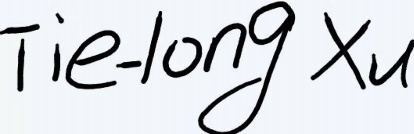


Signature:
